# Editorial: Chronology of gastrointestinal cancers and gastrointestinal microbiota

**DOI:** 10.3389/fendo.2023.1179413

**Published:** 2023-04-13

**Authors:** Yang Mi, Furhan Iqbal, Nasir Mahmood, Ihtisham Bukhari, Pengyuan Zheng

**Affiliations:** ^1^ Henan Key Laboratory of Helicobacter pylori, Microbiota and Gastrointestinal Cancers, Marshall Medical Research Center, Fifth Affiliated Hospital of Zhengzhou University, Zhengzhou, China; ^2^ Institute of Zoology, Bahauddin Zakariya University, Multan, Pakistan; ^3^ Department of Biochemistry, Human Genetics and Molecular Biology, University of Health Sciences, Lahore, Pakistan; ^4^ Department of Cell and Systems Biology, University of Toronto, Toronto, ON, Canada

**Keywords:** chronology, gastrointestinal cancers, gut microbiota, carcinogens, cancer development and metastasis

The term “chronology of cancer” describes a number of factors, including the changing nature of cancers over time, extending from carcinogenesis to development, progression, and metastasis (1, 2). A number of factors can lead to the development of gastrointestinal cancer, including, but not limited to, food, infections, DNA damage, environmental carcinogens, radiation, toxic chemicals, and genetic and epigenetic alterations (3–5). Various chronologies of gastrointestinal cancers have been reported that have brought awareness to the masses and helped to develop cancer prevention strategies. Analyzing tumor growth helps to estimate the time at which cancer or metastasis occurred and to predict the survival of cancer patients (2, 6–8). These chronologies vary from case to case and may even differ for the cancers derived from the same organ (2, 9). Gut microbiota maintains a healthy body and preserves symbiosis with the host immune system that generates protective responses against pathogens and regulatory pathways that sustain tolerance to commensal microbes (10, 11). Recently, the gut microbiota came into the limelight due to their association with multiple cancers, especially gastrointestinal cancers (6, 8, 10). It is already an established fact that chronic *Helicobacter pylori* infection is an essential risk factor for atrophic gastritis, intestinal metaplasia and gastric cancer (12, 13). Recently, it has been highlighted that the gut resident microbiota influences the response to cancer-related therapies (14–16). However, gaps still exist regarding the functional activity and the microbiome changes in the gut during cancer development and progression. Thus, the alterations in the gut microbiota are being monitored to assess the antitumor response of the drugs and the modulation of the intestinal immune system (17, 18). It has been indicated that dysbiosis of gut microbiota and associated metabolites contribute to carcinogenesis through multiple pathways, such as inducing inflammation, immune dysregulation, and genetic instability (19–21). The current Research Topic aims to report the data related to the influence of gut microbiota on the development of gastrointestinal (GI) cancers (including esophageal, gastric, colorectal, liver, and pancreatic cancers) and new strategies for the prevention and treatment of GI cancers. After rigorous screening and reviewing, only four articles exploring new dimensions in this research field were included in the current topic.

Liu et al. studied the safety features of natural orifice specimen extraction (NOSE) in treating 125 patients with the sigmoid colon and upper rectal cancer. Specifically, they focused on the clinical and pathological characteristics, inflammatory and immune indicators, and postoperative complications following NOSE application. Liu et al. divided the patients into two groups based on the treatment they received, Conventional laparoscopic-assisted radical resection for CRC CLA) and laparoscopic-assisted radical resection for CRC with NOSE (La-NOSE) and found no significant difference in post-surgery complications in both groups. Liu et al. suggested that even with a high rate of intra-abdominal bacterial load, NOSE is still safe and an alternative therapeutic option for colorectal cancer.

Cai et al. retrospectively evaluated the Regional Lymph node Metastasis (LNM) pattern to strategize the optimal surgery standpoint of lymph node distribution in 6622 patients that were diagnosed with localized well-differentiated gastroenteropancreatic neuroendocrine tumors (GEP-NETs) that were ≤ 20 mm in size. 36% of patients showed LNM after regional lymphadenectomy. Nodal involvement was observed in different GI cancers at different proportions. It was concluded that patients at a young age with big tumors and muscular invasion had higher LNM and lymphadenectomy can help to increase the overall survival of NETs patients.

Lv et al. found that acupuncture can improve gut function and enhance the microbiota’s quality after chemotherapy in mice. Furthermore, a significant correlation was observed between gut microbiota, inflammation, and serum metabolites. This study opened new doors for basic and clinical research on cancer-related fatigue after cancer therapy.

Wang et al. determined the plasma level of lncRNA lnc-MyD88 in 98 hepatocellular carcinomas (HCC) patients. In addition, they analyzed the relationship of lnc-MyD88 with clinicopathological features and immune cell infiltration in the liver cancer tumor microenvironment. Notably, they found high expression of lnc-MyD88 in HCC and a positive correlation with immune function and suggested it to be used as a diagnostic marker for liver cancer.

## Conclusion and prospects

Gastrointestinal (GI) cancers are ranked among the most frequently reported cancer type worldwide and rated as the third leading cause of cancer-associated deaths. Chemotherapy, immunotherapy, radiation, and surgery are treatment options for various cancers, including GI. Since the chronology of gastrointestinal cancer is not fully explored, we provide some evidence to support the advancements in clinical and basic research, especially in gastroenterology. In the current topic, we selected studies describing the different aspects of GI cancers, including gut microbiota, the role of lncRNAs, Lymph node Metastasis (LNM), and NOSE therapy. Further and advanced studies are required to explain gastrointestinal cancers’ chronology and underlying mechanisms for effective treatment.

## Author contributions

All authors listed have made a substantial, direct, and intellectual contribution to the work and approved it for publication.
